# Supplementary material for: Evidence for a Strong Relationship between the Cytotoxicity and Intracellular Location of β-Amyloid
Source: Life (Basel). 2022 Apr 13;12(4):577. doi: 10.3390/life12040577 (PMC9025630; doi:10.3390/life12040577)
Supplement: Supplementary file 1 [file life-12-00577-s001.zip › life-1612256-supplementary.pdf]

Supplementary Figures

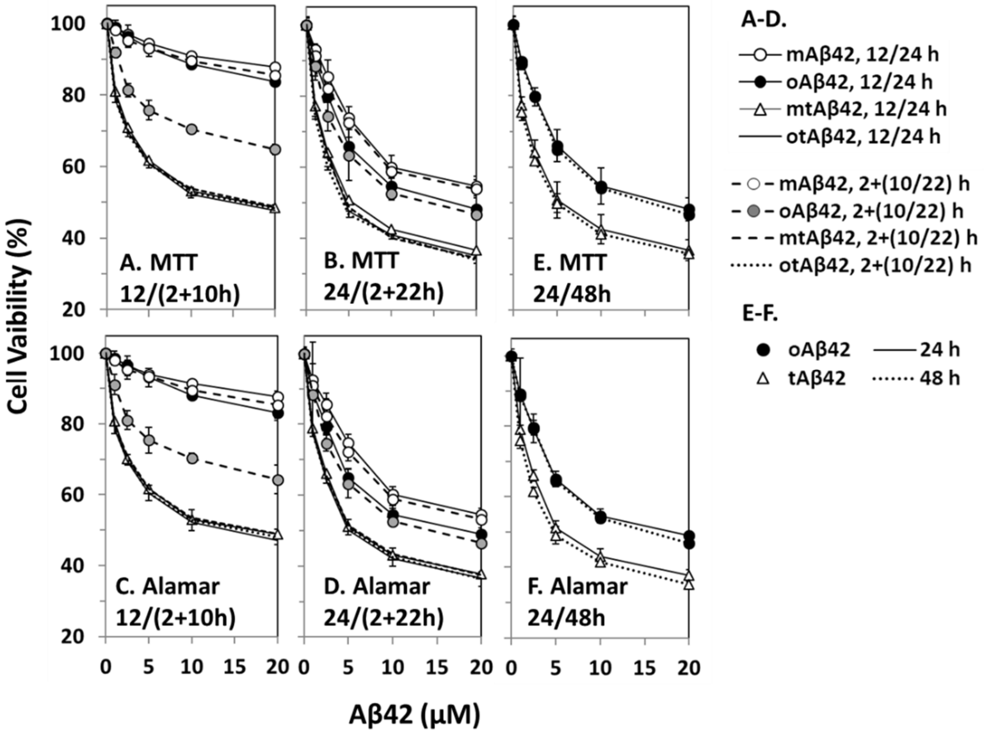

Figure S1. Cytotoxicity of Aβ42 and tAβ42. SH-SY5Y cells were treated with mAβ42, oAβ42, mtAβ42, and otAβ42 at the indicated concentrations for 12/(2+10) h (A, B), 24/(2+22) h (C, D) and 24/48 h (E, F). After treatment, cell viability was assessed with the MTT reduction assay and alamarBlue assay. Results are expressed as the mean ± standard deviation of values from three independent experiments. Data for Aβ42 12 h, mAβ42 2+10 h and oAβ42 12 h in A and B; mtAβ42 12 h, mtAβ42 2+10 h, otAβ42 12 h and otAβ42 2+10 h in A and B; mtAβ42 24 h, mtAβ42 2+22 h, otAβ42 24 h and otAβ42 2+22 h in C and D are overlapped.

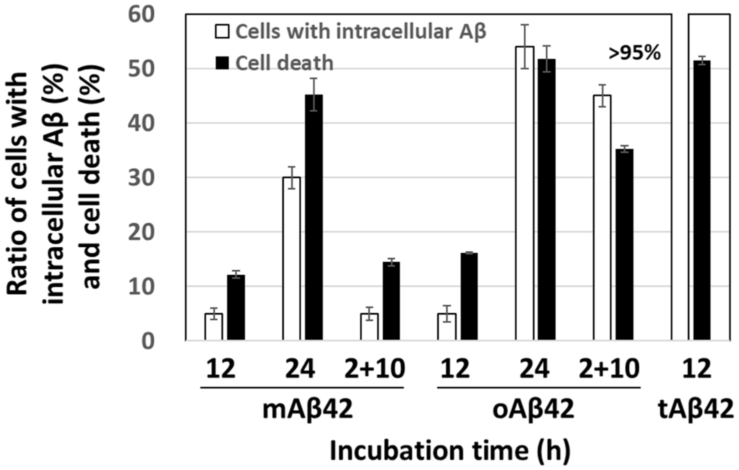

Figure S2. Extent of cellular internalization of Aβ42 and tAβ42. SH-SY5Y cells were treated with Aβ42 species as indicated. Next, the confocal microscopic images of the cells were taken for Aβ and caspase-9 by applying mouse anti-Aβ (6E10) and rabbit anti-caspase-9 (p10) antibodies. The number of cells with intracellular Aβ was calculated using the confocal microscopic images and compared with cell death for both peptides. The data for cell death were obtained from supplementary Figure 1. Data are presented as the mean ± standard deviation of values from three independent experiments.

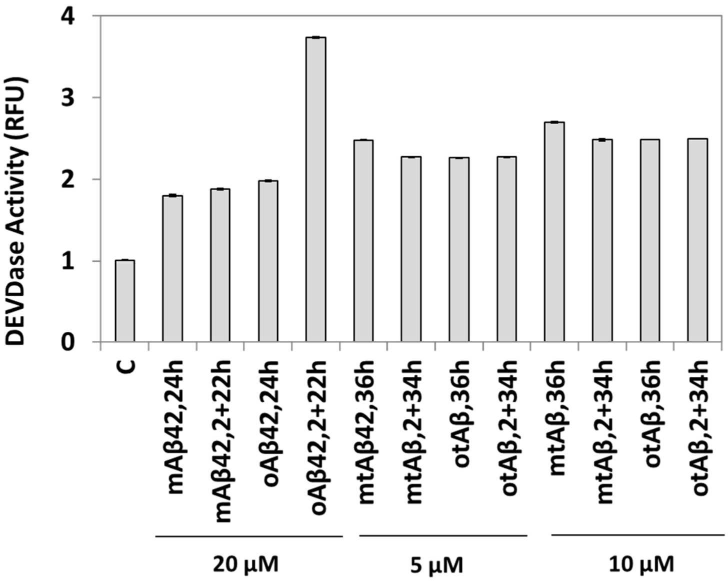

Figure S3. DEVDase activity induced by Aβ42 and tAβ42. SH-SY5Y5 cells were treated with the indicated Aβ42 peptides for the indicated period, and DEVDase activity was measured with 10 μM ac-DEVD-AMC substrate. RFU indicates relative fluorescence unit. Results are expressed as the mean ± standard deviation of values from three independent experiments.
